# Dora Black, MB, DPM, FRCPsych, FRCPCH (Hon.)

**DOI:** 10.1192/bjb.2022.25

**Published:** 2022-12

**Authors:** Philip Graham

Formerly Consultant Child and Adolescent Psychiatrist, Royal Free Hospital, London, and Honorary Consultant Child Psychiatrist, Hospital for Sick Children, Great Ormond Street, London, UK


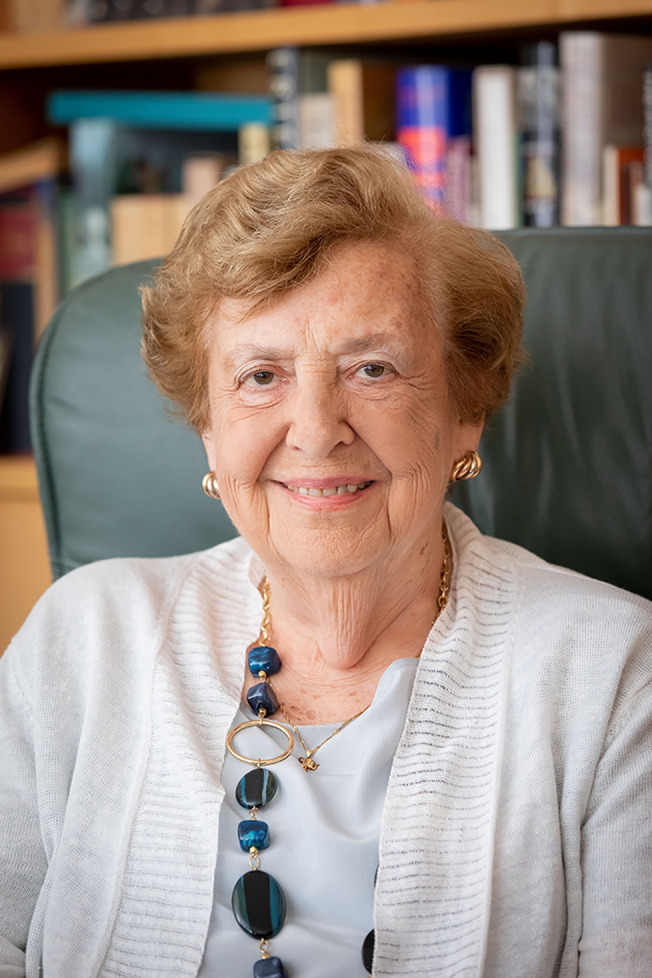


Dora Black, who died on 16 December 2021 aged 89, was a pioneer in the assessment and management of children severely traumatised by the loss of a parent. She began to engage in interventions with bereaved children in the 1970s. This interest was combined with a strong focus on family therapy, then a relatively new approach, and in 1985 she began to publish articles and book chapters on family interventions with bereaved children.

In the 1980s she received a trickle of referrals of children whose father had killed their mother. She developed systematic methods of assessment and intervention for such children. Her trauma clinic at the Royal Free Hospital in London attracted referrals from all over the country of children who had suffered this appalling double tragedy – a double tragedy because not only had they lost a mother through death, but they had also lost their father, now imprisoned. In her clinical work with these children, Dora went more than the extra mile. She went the extra several miles when, for example, she accompanied children on their first visit to their imprisoned fathers. By the time she had retired she had dealt with over 700 such cases. In 1994, together with Jean Harris-Hendriks and Tony Kaplan, she published *When Father Kills Mother*, an account of their approach with these deeply traumatised children.^1^ In 1995, the Royal Free trauma clinic moved to central London to become the Camden Traumatic Stress Clinic, a service that provided for people of all ages, the first of its kind. The clinic being all-age had the great advantage that it could help the whole family and, of course, it is the whole family that is involved when a fatal tragedy occurs, whichever family member dies.

In 1968, at the Edgware General Hospital, Dora became one of the first child psychiatrists to set up ward liaison services with paediatricians. When she moved to the Royal Free Hospital, she was successful in being able to persuade the paediatricians there to agree to such services, which she ran until her retirement. After retirement, she was invited in 1999 to become a member of the Home Office Review of Prison Mother and Baby Units. She made sure that not only the material needs of very young children were met, but their emotional needs as well. Nursery nurses were appointed to all such units.

Dora Black was born in north London on 2 July 1932, the older of the two daughters of Philip Braham, a businessman running a factory making tubular steel furniture and his wife Rachel (née Saetta), a housewife who had previously run a hairdressing salon. At the outbreak of war, Dora was evacuated to New York with her mother and sister, later moving to Canada to join her father, who was stationed there. Returning to England in 1945, she won a place at Hendon County Grammar School, where she met her future husband Jack. She was determined to study medicine, but despite her more than adequate academic credentials, she was rejected by 21 medical schools before being admitted to Birmingham University, where she won a number of undergraduate prizes.

After graduation and junior medical posts, she underwent psychiatric training at the Maudsley Hospital in London (1957–1960). After leaving the Maudsley, she combined bringing up her three children with part-time posts in child psychiatry in Hertfordshire and at the Edgware General Hospital. She was appointed to her first full-time appointment at the Royal Free Hospital in 1984.

She retired from her NHS appointments in 1997, though remaining extremely busy professionally, especially as an expert witness in child custody cases. In 1998, with Stephen Wolkind and Jean Harris-Hendriks, she published the third edition of *Child Psychiatry and the Law*,^2^ a most useful guide for those uninitiated in legal processes that was first published in 1989. After retirement she also continued to serve for a number of years as a consultant to the Rhodes Farm Clinic for children and adolescents with anorexia nervosa.

Dora lectured all over the world on a wide range of topics, but especially on childhood bereavement. She was appointed Visiting Professor at the University of Utah in 1992 and awarded a Winston Churchill Travelling Fellowship to study trauma services in the USA in 1993.

She was a forceful personality whom some people found very challenging. Someone who knew her well rightly said that three words came to mind to best describe her – energy, commitment and tenacity. But to those must be added an essential fourth, caring. She did indeed care very greatly for the children and families who consulted her and showed great compassion towards them.

Dora married Jack Black, a solicitor, in 1955. They shared numerous interests, especially opera, other classical music, walking and travel. He was a constant, loving support to her throughout their long marriage. They had three children, David, who died in 2010, Andrew and Sophie. Jack, Andrew and Sophie survive her, as does a grandson Matthew.
